# Vaginal evisceration related to genital prolapse in premenopausal woman

**DOI:** 10.1590/S1677-5538.IBJU.2016.0249

**Published:** 2017

**Authors:** Lucas Schreiner, Thais Guimarães dos Santos, Christiana Campani Nygaard, Daniele Sparemberger Oliveira

**Affiliations:** 1 Departamento de Obstetrícia e Ginecologia do Hospital São Lucas da Pontifícia Universidade Católica do Rio Grande do Sul, RS, Brasil;; 2 Serviço de Uroginecologia do Hospital São Lucas da Pontifícia Universidade Católica do Rio Grande do Sul, RS, Brasil

**Keywords:** Prolapse, Vagina, Lupus Erythematosus, Systemic

## Abstract

**Background:**

Vaginal evisceration is a rare problem, usually related to a previous hysterectomy. We report a case of spontaneous rupture of the cul-de-sac in a premenopausal woman under treatment with glucocorticoids to treat Systemic Lupus Erythematosus (SLE), with uterine prolapse that occurred during evacuation.

**Case Report:**

A 40-year-old woman with SLE, using glucocorticoids, with uterine prolapse grade 4 (POP-Q), awaiting surgery presented at the emergency room with vaginal bleeding after Valsalva during defaction. Uterine prolapse associated with vaginal evisceration was identified. Under vaginal examination, we confirmed the bowel viability and performed a vaginal hysterectomy and sacrospinous fixation.

**Case hypothesis:**

This case draws attention to the extreme risk of untreated uterine prolapse, as well as the importance of multidisciplinary care of patients with vaginal prolapse and chronic diseases.

## Promising future implications:

Uterine prolapse may be related to serious complications when associated with other chronic diseases.

Vaginal evisceration may occur in premenopausal women, unrelated to vaginal trauma.

## Scenario

Vaginal evisceration is a rare condition, usually related to genital trauma. In the literature reviewed, we found no reference to this occurrence related to genital prolapse without direct genital trauma, as in this case (1-3) (Table-1).

**Table 1:** Prior reports of vaginal evisceration.

First Author/ Year	Clinical cases
Gheewala U, 2015	Transvaginal bowel evisceration in a patient with prolapse, associated with perineal trauma after a fall from a chair.
Matthews C, 2014	Four cases of vaginal cuff evisceration after hysterectomy
Austin JM, 2013	Postcoital vaginal rupture in a 23-year-old woman

Pelvic-organ prolapse is the downward descent of female pelvic organs, which can affect the bladder, uterus, vaginal cuff (post-hysterectomy), and the small or large bowel. Prolapse development is multifactorial; the most consistent risk factors are vaginal delivery, advancing age, increasing body-mass index, and connective-tissue abnormalities (4).

Systemic lupus erythematosus (SLE) is an autoimmune connective-tissue disorder with a wide range of clinical features, which predominantly affects women. SLE can affect the skin, joints, kidneys, central nervous system, serous fluid, blood and immune system. Although glucocorticoids are important in the treatment of SLE, they are related to a number of adverse effects, which vary according to time and dose. The effects on the skin and soft tissues are most prominent (5).

We describe the case of a 40-year-old woman with complete uterine prolapse and SLE, who presented with spontaneous rupture of the vagina during defecation.

## Case Report

**Figure 1:**
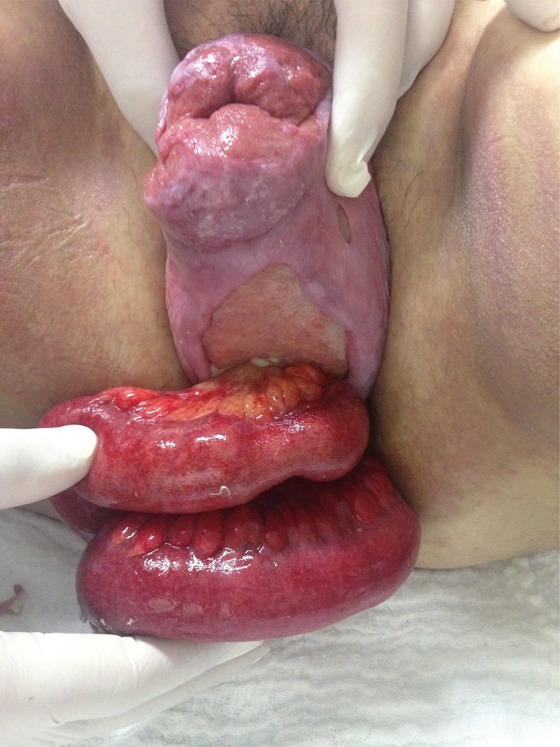
Preoperative.

**Figure 2:**
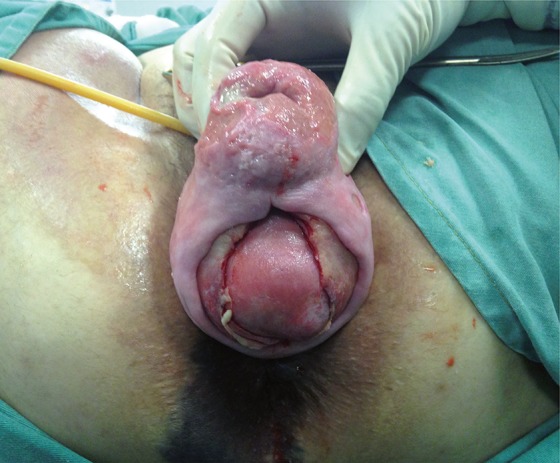
Trans-operative.

A 40 year-old-woman, under treatment for systemic lupus erythematosus for the past 5 years, taking high doses of glucocorticoids without adequate medical supervision, was referred to our department with complete uterine prolapse associated with 2 vaginal ulcers. She had a body-mass index of 31.2kg/m^2^, 3 previous spontaneous vaginal deliveries (last one 3 years before this incident), hypertension under treatment, Cushingoid appearance, and no previous surgeries. After preoperative evaluation, her surgery was scheduled. Fifteen days before the date planned, she presented at the emergency room describing significant vaginal bleeding after Valsalva during defecation. She denied any history of constipation. An examination identified spontaneous rupture of vagina, with evisceration of small bowel loops. The bowel loop herniated through a hernia ring which measured about 5cm, located on the posterior vaginal wall (Figure-1). We immediately covered the bowel with warm sterile gauzes and started antibiotic therapy (Cyprofloxacine and Methronidazole). The patient was taken to the operating room. Initially, the small bowel was assessed vaginally and carefully inspected for areas of devascularization (Figure-2). We performed a manual reduction of the loops and closed the vaginal rupture using unabsorbable sutures (polypropylene). At the same time, vaginal hysterectomy, anterior vaginal repair, posterior vaginal repair and sacrospinous fixation of the vaginal vault at the right sacrospinous ligament were performed. All procedures were performed through the vaginal route. During postoperative follow-up, the patient had good evolution, but she was only discharged seven days after the procedure as she has to compensate her SLE.

## Discussion and future perspectives

This report describes an extremely rare but potentially catastrophic complication related to genital prolapse. We believe that lupus and irregular corticosteroid intake may be associated factors with the vaginal rupture.

Pelvic-organ prolapse is a benign disease, with no relationship to mortality. Most patients with pelvic-organ prolapse are asymptomatic. Seeing or feeling a bulge of tissue that protrudes to or past the vaginal opening is the most specific symptom. This condition may potentially affect millions of women worldwide. Currently, it is the most common non-cancer indication for hysterectomy in menopausal women in the United States (4).

Patients may present with complaints related to prolapse, including bladder, bowel, and pelvic symptoms; however, with the exception of vaginal bulging, none is specific to genital prolapse. Women with symptoms suggestive of prolapse should undergo a pelvic examination and review of medical history. When prolapse is symptomatic, options include observation, pessary use, and surgery (4).

Burge et al. studied the prevalence and pattern of mucosal involvement in 121 patients with lupus erythematosus. Twenty-one per cent of the patients with SLE had signs of mucosal involvement, which could contribute to vaginal weakness (6).

Glucocorticoids are estimated to be used long-term by 0.5–1% of the general population and up to 2.5% of older adults. Chronic use of corticosteroids is related to many side effects (7). Mean plasma estrone levels are significantly low in patients treated with a corticosteroid (8). We therefore hypothesized that the very low plasma estrone levels, which are secondary to pituitary suppression and consequent low androstenedione levels, may have contributed to the weakness of the vaginal mucosa in our patient.

All reports of vaginal evisceration are related to some direct trauma as the causal factor, such as coitus, obstetric, pessary use, or pelvic surgery (1-3). We hypothetized, based on our case, that evisceration can occur even without direct significant trauma, because other factors contribute to weakness of the mucosa.

Atrophy and menopausal status are considered risk factors for evisceration (1, 2). This patient was 40 years old and premenopausal, but she probably had clinically moderate atrophy secondary to corticosteroid use, which may have contributed to the evisceration.

As vaginal evisceration is a gynecological emergency, it is essential to treat this condition promptly, focusing on maintaining bowel viability and reducing the risk of infection though immediate surgical correction. When genital prolapse is present, concomitant management of it can be a good option, reducing the risk of recurrence of the evisceration.

This case illustrates the importance of effective team care to treat patients with chronic diseases. In a severe case like this, when an urgently schedule for surgical treatment is not possible due to clinical conditions, a pessary as intermediate intervention could have helped to prevent the evisceration. It also shows a possible association between SLE, chronic use of a glucocorticoid, and fragility of the vaginal mucosa, causing possible fatal complications in benign pathologies, such as uterine prolapse.
